# Treating Clinically Node-Negative Insular Thyroid Carcinoma without Prophylactic Central Compartment Neck Dissection Is Associated with Decreased Survival Regardless of *T* Staging and Administration of Radioactive Iodine Therapy: The First Evidence

**DOI:** 10.1155/2019/3078012

**Published:** 2019-10-16

**Authors:** Peng-Cheng Yu, Xiao Shi, Ben Ma, Cui-Wei Li, Li-Cheng Tan, Wei-Ping Hu, Yu Wang, Wen-Jun Wei, Yu-Long Wang, Qing-Hai Ji

**Affiliations:** ^1^Department of Head and Neck Surgery, Fudan University Shanghai Cancer Center, Shanghai, 200032, China; ^2^Department of Oncology, Shanghai Medical College, Fudan University, Shanghai, 200032, China; ^3^Department of Internal Medicine, Shanghai Medical College, Fudan University, Shanghai, 200032, China

## Abstract

For the rare but aggressive insular thyroid carcinoma (ITC), there's no clear evidence to determine whether prophylactic central compartment neck dissection (CCND) is necessary for cN0 disease. This study provides the first evidence that treating cN0 ITC without prophylactic CCND is associated with decreased survival regardless of *T* staging and administration of RAI therapy. *Background*. Regarding the rare but aggressive insular thyroid carcinoma (ITC), the value of prophylactic central compartment neck dissection (CCND) for clinically node-negative (cN0) disease is unclear. We aimed to provide the first evidence. *Methods*. N0 and pN1a ITC patients were identified from the Surveillance, Epidemiology, and End Results database. These patients were divided into *thyroid-surgery* + CCND group (pN0/pN1a patients confirmed by CCND) and *thyroid-surgery* group (cN0 patients without CCND). Differences in overall survival (OS) and disease-specific survival (DSS) between the two groups were evaluated. Subgroup analyses were also conducted. *Results*. Of the overall 112 patients, 44 (39.3%) received CCND. On multivariate analyses, the *lobectomy* ± *isthmusectomy*/*total-thyroidectomy* (Lob/TT) group demonstrated poorer OS and DSS than the Lob/TT + CCND group (*P* < 0.05). When we separately analyzed patients treated by TT, multivariate analyses showed the TT group still revealed compromised OS and DSS than the TT + CCND group (*P* < 0.05). Furthermore, absence of CCND independently predicted decreased OS no matter whether radioactive iodine (RAI) was administered. Similar results were obtained for T3/T4 patients. Moreover, for T1/T2 patients receiving CCND, 0/12 died during the study period, while for T1/T2 patients without CCND, 8/23 (34.8%) died, 5/23 (21.7%) due to ITC. *Conclusion*. Regardless of *T* staging and RAI treatment, cN0-ITC patients without CCND had decreased survival compared with pN0/pN1a patients receiving CCND. Therefore, if a cN0 patient is diagnosed with ITC, prophylactic CCND may be considered as a secondary procedure (postoperatively diagnosed) or a primary procedure (preoperatively/intraoperatively diagnosed). Prospective studies are expected to validate the conclusion.

## 1. Introduction

First described by Carcangiu et al. in 1984 [1], insular thyroid carcinoma (ITC) is a rare but aggressive thyroid malignancy categorized as the most common subtype of poorly differentiated thyroid carcinoma (PDTC) [2–4]. Data from both small series and population-level databases show that ITC is associated with larger tumor size, higher rate of extrathyroidal extension (ETE), nodal involvement, and distant metastasis [2, 5–8].

The value of prophylactic central compartment neck dissection (CCND) in clinically node-negative (cN0) differentiated thyroid cancer (DTC) has been a hot topic for decades but is still a matter of debate. With regard to PDTC found postoperatively, the previous National Comprehensive Cancer Network (NCCN) guideline (2017 version 2) lists CCND as a consideration for cN0 patients [9]. However, this statement is deleted in the latest version (2018 version 1) [10]. On the other hand, in the 2015 American Thyroid Association (ATA) guideline, indications of prophylactic CCND for ITC or PDTC are not separately distinguished from classic DTCs [3].

Although the 2015 ATA guideline recommends prophylactic CCND for T3/T4 cN0 patients, lobectomy without CCND is regarded appropriate for cN0 patients with T1/T2 tumors [3]. Besides, several scholars also reported that with the use of radioactive iodine (RAI) ablation, total thyroidectomy without CCND could also achieve a low locoregional recurrence rate or low postoperative thyroglobulin levels in cN0 patients [11, 12].

Due to the aggressive behavior of the insular subtype, whether cN0 ITC necessitates CCND, especially for T1/T2 patients or those undergoing RAI therapy, deserves further attention in clinical practice. However, given the paucity of data, this problem has never been investigated. In this study, we sought to explore this issue using data from the Surveillance, Epidemiology, and End Results (SEER) database.

## 2. Materials and Methods

### 2.1. Data Source and Study Population

Using the SEER database, the eligible patients were identified according to the following selection criteria: (1) diagnosed with ITC as the first malignancy using the International Classification of Diseases for Oncology, 3rd edition (ICD-O-3) code 8337; (2) diagnosed between 1999 and 2014, as the first ITC patient recorded in the SEER program was diagnosed in 1999; (3) received thyroid surgery; (4) without distant metastasis at diagnosis; (5) with N0 or pN1a record of diagnosis; (6) with definite record of *T* staging and known tumor size. We excluded patients without information on whether CCND was performed and those less than 18 years. The selection process is presented as a flow diagram (Supplementary [Supplementary-material supplementary-material-1]). In total, 112 patients were identified and constituted the study cohort.

Institutional Review Board (IRB) approval is not necessary for this study, because the SEER program is a publicly available cancer database with deidentified data.

### 2.2. Variables and Outcomes

Data extracted for each case included age at diagnosis, sex, race, *T* and *N* stages, multifocality, extrathyroidal extension (ETE), tumor size, thyroid surgery, radiation, and presence/absence of CCND. As small ITCs were sometimes found after lobectomy, in the present study, thyroid surgery included both *lobectomy* *±* *isthmusectomy* (Lob) and *total thyroidectomy* (TT) to better reflect the real clinical situation. Radiation was divided into three categories: “no evidence,” “external beam radiotherapy (EBRT),” and “RAI,” just like the previous studies [2, 5]. As only four patients received chemotherapy, this parameter was not included in the analyses. The *T* and *N* staging were identified according to the American Joint Committee (AJCC) eighth edition.

The N0 patients included pN0 patients confirmed by CCND and cN0 patients without prophylactic CCND. It is worth noting that, in the SEER database, if a patient has a pathologic stage, the record of clinical stage will be covered and not available to users. Consequently, the cN staging (cN0/cN1) of pN0 and pN1a patients was unknown, the CCND of whom might include both prophylactic CCND and therapeutic CCND. Subsequently, the overall cohort was divided into the *thyroid-surgery* + CCND group (pN0/pN1a patients confirmed by CCND) and *thyroid-surgery* group (cN0 patients without CCND).

Overall survival (OS) and disease-specific survival (DSS), respectively, defined as the interval from initial pathologic diagnosis to the date of all-cause or ITC-specific death were compared between the two groups.

### 2.3. Statistical Analysis

The differences of baseline characteristics across the two groups were compared by chi-squared test or Fisher's exact test, as appropriate. The survival curves were plotted by Kaplan––Meier estimates, and the differences were compared by log-rank tests. Multivariate Cox proportional hazards regression models were employed to identify the prognostic factors after adjusting for potential confounders, and the results were presented as hazard ratios (HRs) with 95% confidence intervals (CI). To better evaluate the prognostic relevance of CCND, for all multivariate analyses (MVA), two types of Cox regression models were parallelly performed. The Type-A model was defined as the Cox regression model including comprehensive baseline variables (age at diagnosis, sex, race, multifocality, ETE, tumor size, thyroid surgery, radiation, and CCND), *T* stage was not incorporated because the two factors, tumor size and ETE, which constituted the *T* staging were both included in the model. The Type-B model included the best subsets of covariates identified by the smallest Akaike Information Criterion (AIC) value, which reflected the minimal loss of information [13–15].

Log-rank tests and multivariate Cox analyses were conducted by SPSS Statistics v23.0 (SPSS Inc., Chicago, IL). Kaplan–Meier survival plots were generated by GraphPad Prism 7.0 (GraphPad Software, San Diego, CA). The AIC values were calculated by R version 3.4.3 (R-Foundation for Statistical Computing, Vienna, Austria). Statistical significance was defined as a two-tailed *P* value < 0.05.

## 3. Results

### 3.1. Patient Characteristics

Of the 112 patients, the median follow-up length was 67 months (range: 2–167 months) and the median age at diagnosis was 58 years (range: 21–94). 64.3% of patients (*n* = 72) had tumors larger than 4 cm, nearly three fourths of the tumors were unifocal (*n* = 83, 74.1%), and approximately one third (*n* = 38, 33.9%) had ETE. 98 (87.5%) patients underwent TT, and 70 (62.5%) received RAI therapy (Table 1).

102 patients were recorded as N0, 34 were pN0 receiving CCND, and 68 patients were cN0 not receiving CCND. 10 patients were diagnosed with pN1a disease (possible reasons were discussed in Discussion), and none of these patients had level VII (upper mediastinal) metastases. The pN0 and pN1a patients undergoing CCND constituted the Lob/TT + CCND group (*n* = 44). The 68 cN0 patients without CCND constituted the Lob/TT group. Baseline characteristics across the two groups are also shown in Table 1. A significantly higher proportion of patients with ETE was observed in the Lob/TT + CCND group (*P*=0.038).

### 3.2. CCND versus No-CCND in the Overall Cohort

**Figure 1:**
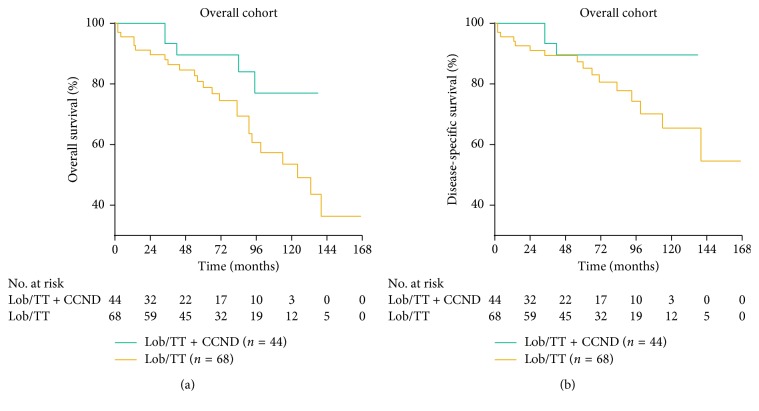
Kaplan–Meier survival plots presenting (a) OS and (b) DSS of the Lob/TT group and Lob/TT + CCND group for patients in the overall study cohort (99 × 43 mm (300 × 300DPI)).

The survival difference between the Lob/TT + CCND group and the Lob/TT group was investigated for the overall cohort. Among 44 patients in the Lob/TT + CCND group, 5 (11.4%) died during the study period, 3 (6.8%) of which were due to ITC. Among 68 patients in the Lob/TT group, we observed 25 (35.8%) deaths and 16 (23.5%) were due to ITC (Figure 1). The log-rank test showed that the Lob/TT + CCND group had a significantly improved OS compared with the Lob/TT group (*P*=0.048). No significant difference in DSS was observed in the log-rank test (*P*=0.078).

As we mentioned in Materials and Methods, two types of MVA were performed. (i) In the Type-A Cox models, the Lob/TT group was independently associated with worse OS (HR = 4.640 (95% CI 1.576–13.659), *P*=0.005) and DSS (HR = 5.707 (95% CI 1.362–23.907), *P*=0.017) compared with the Lob/TT + CCND group (Table 2). (ii) The smallest AIC values (214.9 for OS; 143.3 for DSS) were obtained when we incorporated age at diagnosis, sex, multifocality, ETE, and CCND into the Type-B Cox models for both endpoints; the Lob/TT group still had significantly poorer prognosis (OS : HR = 4.408 (95% CI 1.510–12.865), *P*=0.007; DSS : HR = 5.523 (95% CI 1.342–22.730), *P*=0.018) (Supplementary [Supplementary-material supplementary-material-1]).

### 3.3. CCND versus No-CCND for Patients Treated with TT

Subsequently, we focused on patients treated with TT. (i) In the Type-A Cox models, the TT group demonstrated significantly inferior OS (HR = 4.175 (95% CI 1.325–13.158), *P*=0.015) and DSS (HR = 5.087 (95% CI 1.089–23.751), *P*=0.039) compared with the TT + CCND group (Table 3). (ii) In the Type-B models, the significantly higher mortality risk of the TT group was also observed (OS : HR = 4.242 (95% CI 1.376–13.082), *P*=0.012; DSS : HR = 5.272 (95% CI 1.170–23.767), *P*=0.030) (Supplementary [Supplementary-material supplementary-material-1]).

### 3.4. CCND versus No-CCND according to Whether RAI Therapy Was Administered

For patients who underwent TT and RAI, absence of CCND still predicted compromised survival in both Type-A (OS : HR = 7.137 (95% CI 1.354–37.636), *P*=0.021; DSS : HR = 15.796 (95% CI 1.380–180.789), *P*=0.026) and Type-B Cox models incorporating age at diagnosis, multifocality, ETE, and CCND determined by the smallest AIC (OS : HR = 7.350 (95% CI 1.440–37.520), *P*=0.016; DSS : HR = 14.933 (95% CI 1.442–154.649), *P*=0.023), as shown in Supplementary Tables [Supplementary-material supplementary-material-1] and [Supplementary-material supplementary-material-1].

For patients who did not receive RAI ablation, the prognostic difference failed to meet statistical significance in Type-A Cox regression models (OS : HR = 3.951 (95% CI 0.803–19.945), *P*=0.082; DSS : HR = 2.977 (95% CI 0.786–15.539), *P*=0.095) (Supplementary [Supplementary-material supplementary-material-1]). However, absence of CCND still turned out to be an independent adverse prognostic factor for OS (OS : HR = 3.972 (95% CI 1.103–14.155), *P*=0.035) in the Type-B model controlling for variables identified by the smallest AIC, as shown in Supplementary [Supplementary-material supplementary-material-1].

### 3.5. CCND versus No-CCND according to *T* Stage

For patients with T3/T4 disease, similar results were obtained. As shown in Supplementary Tables [Supplementary-material supplementary-material-1] and [Supplementary-material supplementary-material-1], both the two types of MVA confirmed the increased risk of mortality for patients without CCND (Type-A : OS: HR = 3.659 (95% CI 1.196–11.196), *P*=0.023; DSS : HR = 4.414 (95% CI 1.084–17.967), *P*=0.038; Type-B : OS: HR = 3.318 (95% CI 1.107–9.946), *P*=0.032; DSS : HR = 4.178 (95% CI 1.040–16.780), *P*=0.044).

**Figure 2:**
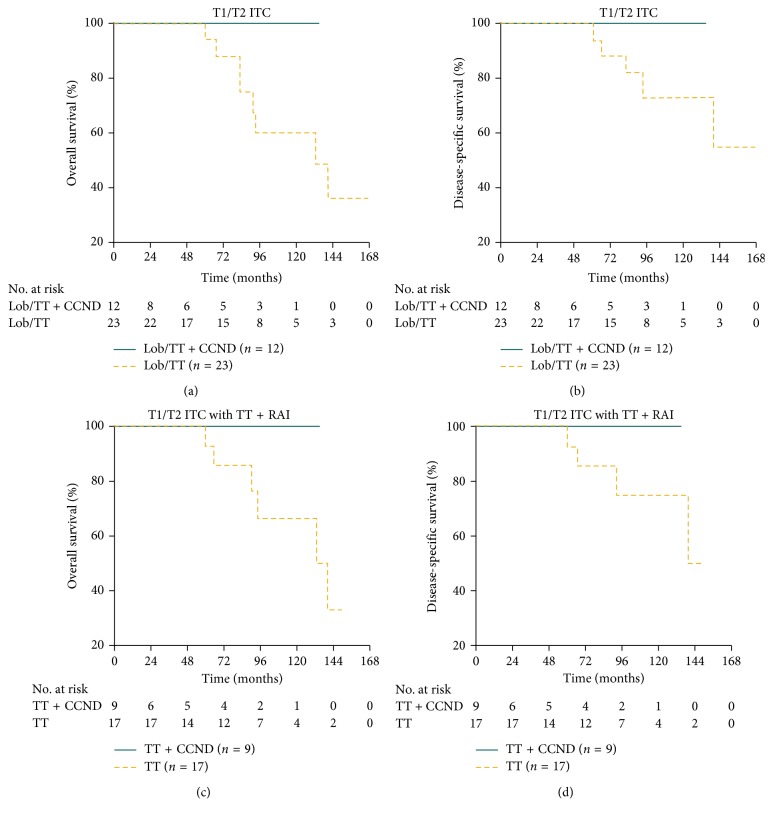
Kaplan–Meier survival plots presenting (a) OS and (b) DSS of the Lob/TT group and Lob/TT + CCND group for T1/T2 patients and (c) OS and (d) DSS of the TT group and TT + CCND group for T1/T2 patients treated with TT and RAI (99 × 87 mm (300 × 300 DPI)).

For patients with T1/T2 disease, there were no significant differences in age at diagnosis, sex, race, multifocality, thyroid surgery, and radiation (Supplementary [Supplementary-material supplementary-material-1]). However, during the study period with a median follow-up time of 83 months (range: 3–167 months), none of the 12 patients died (0/12, 0.0%) in the Lob/TT + CCND group, while 8/23 (34.8%) died in the Lob/TT group and 5 (21.7%) were due to ITC (Figures 2(a) and 2(b)). Multivariate analyses could not be performed because there was no death in the Lob/TT + CCND group.

Furthermore, we focused on T1/T2 patients undergoing TT plus RAI and all-cause mortality in the TT + CCND group and TT group was 0/9 (0.0%) and 6/17 (35.3%), respectively, while disease-specific mortality in the two groups was 0/9 (0.0%) and 4/17 (23.5%), respectively (Figures 2(c) and 2(d)). Similarly, MVA could not be conducted due to the lack of death (*n* = 0) in the TT + CCND group.

## 4. Discussion

Biologically and morphologically, ITC occupies an intermediate position between well-differentiated thyroid carcinoma (WDTC) and anaplastic (undifferentiated) thyroid carcinoma (ATC) [2]. Kazaure et al. firstly reported that the insular subtype was independently associated with a poor survival compared to WDTC using the SEER database [5]. Pezzi et al. reported the 5- and 10-year survival rates of ITC were, respectively, 57% and 30%, significantly worse than papillary thyroid cancer (PTC) with the 5- and 10-year survival rates of 94% and 88% using the National Cancer Database (NCDB) [2].

Nevertheless, neither the 2015 ATA nor the latest NCCN guideline pays special attention to the indication of prophylactic CCND for ITC patients [3, 10], possibly due to the fact that no previous research has provided direct evidence. Owing to the rarity of ITC, most published literatures are case reports or small institution-based case series. Studies with relatively sufficient number of cases rely more on population-level databases. The SEER program is a nationwide cancer database currently covering approximately 34.6 percent of the U.S. population [16]. More importantly, this database is available to cancer researchers worldwide, making it an important tool for clinical research across the globe, especially for rare tumors.

Although Conzo et al. reported that TT without routine prophylactic CCND, followed by RAI ablation also revealed low recurrence rate [11], we found that even with adjuvant RAI therapy, cN0 ITC patients without CCND still have significantly worse prognosis, suggesting that RAI ablation could not replace the therapeutic value of CCND in the treatment of this aggressive subtype. The generally low avidity for RAI in ITC might be a possible explanation. In a French cohort consisting of 104 PDTC patients (nearly 90% were ITC), 50% (*n* = 52) were identified to be RAI-refractory [17]. For patients receiving TT and RAI, absence of CCND demonstrated approximately 7- and 15-fold increased risk of all-cause and disease-specific mortality in the Type-A and Type-B Cox models, respectively. We assumed that such high HRs was caused by the small number of deaths (3 all-cause deaths, 1 due to ITC) among those receiving TT, RAI, and CCND.

Another important result derives from the subgroup analyses for T1/T2 disease. Although *P* values of less than 0.05 could not be achieved because of the small samples, the non-negligible difference in the number of deaths between T1/T2 patients with and without CCND also prompted us to attach importance to the value of CCND in early-stage ITC patients.

As a most common subtype of PDTC, accurate preoperative diagnosis of ITC is crucial for planning optimal surgical management. However, its cytomorphological features are not well characterized, making it easily to be missed in fine-needle aspiration biopsy (FNAB) [18]. At present, it is mostly diagnosed by the pathology report of thyroidectomy specimens. Although Kane and Sharma tried to define the cytopathological features of PDTC in FNAB, more collaborative efforts are necessary to develop standardized cytologic diagnostic criteria [18]. It was also reported that the next-generation sequencing approach was useful to detect genetic biomarkers of PDTC and ATC [19]. However, this technique had a distance to go before its wide use in clinical practice.

A noteworthy point is that there are only 10 eligible pN1a patients in our study cohort despite the aggressive behavior of ITC. In a study using NCDB data, only 27 of 405 M0 ITC cases were diagnosed with N1a disease [2]. When we reviewed our selection process shown in Supplementary [Supplementary-material supplementary-material-1], we found a higher proportion of patients were recorded as “M1/MX” (*n* = 43) or “N1b/N1-NOS/NX” (*n* = 33), suggesting that ITC tends to involve more extensive regions and disease confined to the central compartment is not as common as it is in PTC.

Some limitations should be acknowledged. First, as mentioned above, whether the pN0/pN1a patients were cN0 or cN1 was unknown in SEER database, so some patients in the *thyroid-surgery* + CCND group might undergo therapeutic CCND. Second, surgical margins were not recorded, although the *thyroid-surgery* + CCND group revealing better prognosis had a significantly higher proportion of patients with extrathyroidal extension, which sometimes lead to a difficulty in R0 resection. Third, dose of RAI, BRAF mutational status, and comorbidities were not provided in the database. Fourth, age at diagnosis of the CCND and No-CCND groups was not totally the same, which possibly influenced prognosis to some extent. However, in this study, the various multivariate Cox analyses for the overall cohort or subgroups might reduce the confounding effect of patients' age to a large extent. Fifth, this study is limited by its retrospective nature.

In conclusion, treating cN0 ITC without CCND is associated with decreased survival compared with pN0/pN1a patients receiving CCND irrespective of *T* staging and administration of adjuvant RAI therapy. Therefore, if a cN0 patient is diagnosed as ITC, after fully weighing the benefits and risks, prophylactic CCND may be considered as a secondary procedure (if postoperatively diagnosed) or a primary procedure (if intraoperatively/preoperatively diagnosed), especially for experienced high-volume surgeons. If possible, prospective studies are expected to validate our findings.

## Figures and Tables

**Table 1:** Baseline characteristics of the overall cohort (*N* = 112).

Characteristics	Overall cohort *n* = 112	Lob/TT + CCND group *N* = 44	Lob/TT group *N* = 68	*P*
Age at diagnosis				0.360
Median (range)	58 (21–94)	55 (21–94)	60 (21–88)	
<55	45 (40.2%)	20 (45.5%)	25 (36.8%)	
≥55	67 (59.8%)	24 (54.5%)	43 (63.2%)	
Sex				0.174
Female	65 (58.0%)	29 (65.9%)	36 (52.9%)	
Male	47 (42.0%)	15 (34.1%)	32 (47.1%)	
Race				0.703
White	87 (77.7%)	34 (77.3%)	53 (77.9%)	
Black	15 (13.4%)	5 (11.4%)	10 (14.7%)	
Others	10 (8.9%)	5 (11.4%)	5 (7.4%)	
Tumor size				0.773
≤4 cm	40 (35.7%)	15 (34.1%)	25 (36.8%)	
>4 cm	72 (64.3%)	29 (65.9%)	43 (63.2%)	
Multifocal				0.423
No	83 (74.1%)	34 (77.3%)	49 (72.1%)	
Yes	19 (17.0%)	8 (18.2%)	11 (16.2%)	
Unknown	10 (8.9%)	2 (4.5%)	8 (11.8%)	
Extrathyroidal extension				0.038
No	74 (66.1%)	24 (54.5%)	50 (73.5%)	
Yes	38 (33.9%)	20 (45.5%)	18 (26.5%)	
AJCC 8th *T* staging				0.465
T1/T2	35 (31.3%)	12 (27.3%)	23 (33.8%)	
T3/T4	77 (68.7%)	32 (72.7%)	45 (66.2%)	
Surgery of thyroid gland				0.380
Lob	14 (12.5%)	4 (9.1%)	10 (14.7%)	
TT	98 (87.5%)	40 (90.9%)	58 (85.3%)	
Radiation				0.475
RAI	70 (62.5%)	30 (68.2%)	40 (58.8%)	
EBRT	12 (10.7%)	3 (6.8%)	9 (13.2%)	
No evidence	30 (26.8%)	11 (25.0%)	19 (27.9%)	

Lob refers to lobectomy ± isthmusectomy; TT = total thyroidectomy; CCND = central compartment neck dissection; EBRT = external beam radiotherapy; RAI = radioactive iodine.

**Table 2:** Type-A multivariate Cox regression models investigating the factors associated with OS and DSS in the overall cohort (*N* = 112).

Variables	OS		DSS	
	HR (95% CI)	*P*	HR (95% CI)	*P*
Age at diagnosis				
<55	Ref		Ref	
≥55	7.663 (2.386–24.607)	0.001	6.630 (1.557–28.234)	0.011
Sex				
Female	Ref		Ref	
Male	2.619 (1.120–6.123)	0.026	2.798 (0.899–8.707)	0.076
Race				
White	Ref		Ref	
Black	0.897 (0.225–3.578)	0.877	2.322 (0.527–10.221)	0.265
Others	0.869 (0.191–3.965)	0.856	1.158 (0.230–5.841)	0.859
Tumor size				
≤4 cm	Ref		Ref	
>4 cm	1.348 (0.553–3.279)	0.512	1.799 (0.564–5.747)	0.321
Multifocal				
No	Ref		Ref	
Yes	3.783 (1.214–11.791)	0.022	6.795 (1.565–29.496)	0.011
Unknown	2.579 (0.717–9.274)	0.147	1.535 (0.314–7.516)	0.597
Extrathyroidal extension				
No	Ref		Ref	
Yes	5.602 (2.302–13.635)	<0.001	11.996 (3.374–42.652)	<0.001
Surgery of thyroid gland				
Lob	Ref		Ref	
TT	0.567 (0.160–2.013)	0.380	0.412 (0.081–2.100)	0.286
Radiation				
RAI	Ref		Ref	
EBRT	0.503 (0.111–2.291)	0.375	0.955 (0.185–4.935)	0.956
No evidence	1.825 (0.692–4.812)	0.224	3.542 (0.959–13.079)	0.058
CCND				
Yes	Ref		Ref	
No	4.640 (1.576–13.659)	0.005	5.707 (1.362–23.907)	0.017

OS = overall survival; DSS = disease-specific survival; HR = hazard ratio; CI = confidence interval; Ref = reference.

**Table 3:** Type-A multivariate Cox regression models investigating the factors associated with OS and DSS for patients treated with TT (*N* = 98).

Variables	OS		DSS	
	HR (95% CI)	*P*	HR (95% CI)	*P*
Age at diagnosis				
<55	Ref		Ref	
≥55	7.794 (1.977–30.724)	0.003	9.541 (1.472–61.830)	0.018
Sex				
Female	Ref		Ref	
Male	2.698 (1.032–7.056)	0.043	3.408 (0.935–12.421)	0.063
Race				
White	Ref		Ref	
Black	1.092 (0.234–5.098)	0.911	3.489 (0.601–20.250)	0.164
Others	1.019 (0.218–4.759)	0.981	1.413 (0.266–7.508)	0.685
Tumor size				
≤4 cm	Ref		Ref	
>4 cm	1.129 (0.411–3.096)	0.815	1.938 (0.508–7.407)	0.333
Multifocal				
No	Ref		Ref	
Yes	3.984 (1.226–12.947)	0.022	8.837 (1.820–42.913)	0.007
Unknown	3.716 (0.817–16.911)	0.089	3.052 (0.376–24.808)	0.297
Extrathyroidal extension				
No	Ref		Ref	
Yes	4.264 (1.662–10.940)	0.003	8.427 (2.089–34.003)	0.003
Radiation				
RAI	Ref		Ref	
EBRT	0.931 (0.198–4.373)	0.928	2.394 (0.430–13.334)	0.319
No evidence	1.392 (0.501–3.869)	0.525	2.720 (0.660–11.214)	0.166
CCND				
Yes	Ref		Ref	
No	4.175 (1.325–13.158)	0.015	5.087 (1.089–23.751)	0.039

## Data Availability

The data used to support the findings of this study are included within the article and within the supplementary information files.

## Supplementary Materials

Supplementary Materials Supplementary Figure S1: flow diagram representing the selection process. Supplementary Table S1: Type-B multivariate Cox regression models investigating the factors associated with OS and DSS in the overall cohort (*N* = 112). Supplementary Table S2: Type-B multivariate Cox regression models investigating the factors associated with OS and DSS for patients treated with TT (*N* = 98). Supplementary Table S3: Type-A multivariate Cox regression models investigating the factors associated with OS and DSS for patients treated with TT + RAI (*N* = 61). Supplementary Table S4: Type-B multivariate Cox regression models investigating the factors associated with OS and DSS for patients treated with TT + RAI (*N* = 61). Supplementary Table S5: Type-A multivariate Cox regression models investigating the factors associated with OS and DSS for patients treated without RAI therapy (*N* = 42). Supplementary Table S6: Type-B multivariate Cox regression models investigating the factors associated with OS and DSS for patients treated without RAI therapy (*N* = 42). Supplementary Table S7: Type-A multivariate Cox regression models investigating the factors associated with OS and DSS for patients with T3/T4-stage (*N* = 77). Supplementary Table S8: Type-B multivariate Cox regression models investigating the factors associated with OS and DSS for patients with T3/T4-stage (*N* = 77). Supplementary Table S9: baseline characteristics of patients with T1/T2 tumors (*N* = 35).
