# Supplementary material for: Treating Clinically Node-Negative Insular Thyroid Carcinoma without Prophylactic Central Compartment Neck Dissection Is Associated with Decreased Survival Regardless of T Staging and Administration of Radioactive Iodine Therapy: The First Evidence
Source: Int J Endocrinol. 2019 Oct 16;2019:3078012. doi: 10.1155/2019/3078012 (PMC6815995; doi:10.1155/2019/3078012)
Supplement: Supplementary Materials — Supplementary Figure S1: flow diagram representing the selection process. Supplementary Table S1: Type-B multivariate Cox regression models investigating the factors associated with OS and DSS in the overall cohort (N = 112). Supplementary Table S2: Type-B multivariate Cox regression models investigating the factors associated with OS and DSS for patients treated with TT (N = 98). Supplementary Table S3: Type-A multivariate Cox regression models investigating the factors associated with OS and DSS for patients treated with TT + RAI (N = 61). Supplementary Table S4: Type-B multivariate Cox regression models investigating the factors associated with OS and DSS for patients treated with TT + RAI (N = 61). Supplementary Table S5: Type-A multivariate Cox regression models investigating the factors associated with OS and DSS for patients treated without RAI therapy (N = 42). Supplementary Table S6: Type-B multivariate Cox regression models investigating the factors associated with OS and DSS for patients treated without RAI therapy (N = 42). Supplementary Table S7: Type-A multivariate Cox regression models investigating the factors associated with OS and DSS for patients with T3/T4-stage (N = 77). Supplementary Table S8: Type-B multivariate Cox regression models investigating the factors associated with OS and DSS for patients with T3/T4-stage (N = 77). Supplementary Table S9: baseline characteristics of patients with T1/T2 tumors (N = 35). [file 3078012.f1.docx]

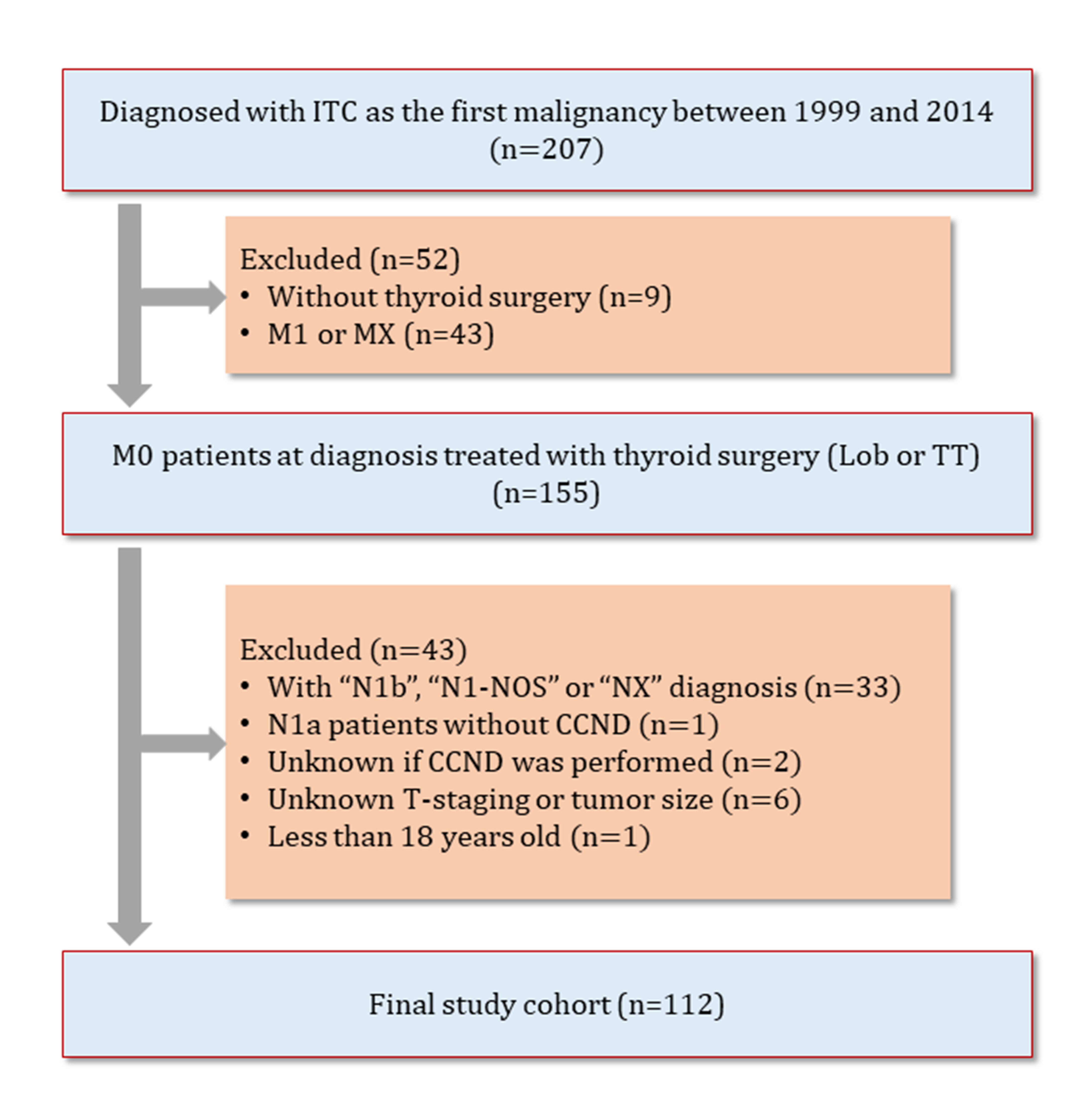


**Supplementary Figure S1:** Flow diagram representing the selection process.

| **Supplementary Table S1: Type B multivariate Cox regression models investigating the factors associated with OS and DSS in the overall cohort (N=112)** | | | | | |
| --- | --- | --- | --- | --- | --- |
| **Variables** | **OS** | |  | **DSS** | |
|  | **HR (95%CI)** | ***P*** |  | **HR (95%CI)** | ***P*** |
| **Age at diagnosis** |  |  |  |  |  |
| <55 | Ref |  |  | Ref |  |
| ≥55 | 5.089 (1.912-13.542) | 0.001 |  | 4.252 (1.211-14.929) | 0.024 |
| **Sex** |  |  |  |  |  |
| Female | Ref |  |  | Ref |  |
| Male | 2.423 (1.102-5.329) | 0.028 |  | 2.155 (0.800-5.806) | 0.129 |
| **Multifocal** |  |  |  |  |  |
| No | Ref |  |  | Ref |  |
| Yes | 3.424 (1.140-10.284) | 0.028 |  | 5.256 (1.392-19.846) | 0.014 |
| Unknown | 2.574 (0.903-7.339) | 0.077 |  | 2.830 (0.731-10.949) | 0.132 |
| **Extrathyroidal extension** |  |  |  |  |  |
| No | Ref |  |  | Ref |  |
| Yes | 4.354 (1.952-9.713) | <0.001 |  | 7.019 (2.417-20.379) | <0.001 |
| **CCND** |  |  |  |  |  |
| Yes | Ref |  |  | Ref |  |
| No | 4.408 (1.510-12.865) | 0.007 |  | 5.523 (1.342-22.730) | 0.018 |

| **Supplementary Table S2: Type B multivariate Cox regression models investigating the factors associated with OS and DSS for patients treated with TT (N=98)** | | | | | |
| --- | --- | --- | --- | --- | --- |
| **Variables** | **OS** | |  | **DSS** | |
|  | **HR (95%CI)** | ***P*** |  | **HR (95%CI)** | ***P*** |
| **Age at diagnosis** |  |  |  |  |  |
| <55 | Ref |  |  | Ref |  |
| ≥55 | 7.210 (1.977-26.296) | 0.003 |  | 5.707 (1.076-30.261) | 0.041 |
| **Sex** |  |  |  |  |  |
| Female | Ref |  |  | Ref |  |
| Male | 2.600 (1.089-6.206) | 0.031 |  | 2.715 (0.876-8.417) | 0.084 |
| **Multifocal** |  |  |  |  |  |
| No | Ref |  |  | Ref |  |
| Yes | 3.926 (1.254-12.294) | 0.019 |  | 6.870 (1.667-28.304) | 0.008 |
| Unknown | 4.373 (1.101-17.362) | 0.036 |  | 3.653 (0.552-24.180) | 0.179 |
| **Extrathyroidal extension** |  |  |  |  |  |
| No | Ref |  |  | Ref |  |
| Yes | 3.893 (1.625-9.326) | 0.002 |  | 6.135 (1.849-20.360) | 0.003 |
| **CCND** |  |  |  |  |  |
| Yes | Ref |  |  | Ref |  |
| No | 4.242 (1.376-13.082) | 0.012 |  | 5.272 (1.170-23.767) | 0.030 |

| **Supplementary Table S3: Type A multivariate Cox regression models investigating the factors associated with OS and DSS for patients treated with TT+RAI (N=61)** | | | | | |
| --- | --- | --- | --- | --- | --- |
| **Variables** | **OS** | |  | **DSS** | |
|  | **HR (95%CI)** | ***P*** |  | **HR (95%CI)** | ***P*** |
| **Age at diagnosis** |  |  |  |  |  |
| <55 | Ref |  |  | Ref |  |
| ≥55 | 6.627 (1.139-38.543) | 0.035 |  | 6.739 (0.881-51.560) | 0.066 |
| **Sex** |  |  |  |  |  |
| Female | Ref |  |  | Ref |  |
| Male | 2.379 (0.694-8.156) | 0.168 |  | 1.265 (0.259-6.191) | 0.771 |
| **Race** |  |  |  |  |  |
| White | Ref |  |  | Ref |  |
| Black | 0.964 (0.183-5.069) | 0.965 |  | 3.424 (0.458-25.606) | 0.231 |
| Other | 2.370 (0.207-27.127) | 0.488 |  | 1.640 (0.082-32.631) | 0.746 |
| **Tumor size** |  |  |  |  |  |
| ≤4 cm | Ref |  |  | Ref |  |
| >4 cm | 2.964 (0.564-15.586) | 0.199 |  | 1.175 (0.115-11.905) | 0.636 |
| **Multifocal** |  |  |  |  |  |
| No | Ref |  |  | Ref |  |
| Yes | 9.402 (1.891-46.753) | 0.006 |  | 26.679 (3.073-231.604) | 0.003 |
| Unknown | 22.213 (2.296-214.937) | 0.007 |  | 9.007 (0.226-359.714) | 0.243 |
| **Extrathyroidal extension** |  |  |  |  |  |
| No | Ref |  |  | Ref |  |
| Yes | 4.415 (1.102-17.679) | 0.036 |  | 9.058 (0.863-95.039) | 0.066 |
| **CCND** |  |  |  |  |  |
| Yes | Ref |  |  | Ref |  |
| No | 7.137 (1.354-37.636) | 0.021 |  | 15.796 (1.380-180.789) | 0.026 |

| **Supplementary Table S4:Type B multivariate Cox regression models investigating the factors associated with OS and DSS for patients treated with TT+RAI (N=61)** | | | | | |
| --- | --- | --- | --- | --- | --- |
| **Variables** | **OS** | |  | **DSS** | |
|  | **HR (95%CI)** | ***P*** |  | **HR (95%CI)** | ***P*** |
| **Age at diagnosis** |  |  |  |  |  |
| <55 | Ref |  |  | Ref |  |
| ≥55 | 4.628 (0.712-30.080) | 0.109 |  | 4.432 (0.478-41.072) | 0.190 |
| **Multifocal** |  |  |  |  |  |
| No | Ref |  |  | Ref |  |
| Yes | 6.541 (1.509-28.344) | 0.012 |  | 19.887 (2.742-144.242) | 0.003 |
| Unknown | 10.045 (1.028-98.110) | 0.047 |  | 8.425 (0.393-180.582) | 0.173 |
| **Extrathyroidal extension** |  |  |  |  |  |
| No | Ref |  |  | Ref |  |
| Yes | 6.609 (1.784-24.484) | 0.005 |  | 9.010 (1.357-59.844) | 0.023 |
| **CCND** |  |  |  |  |  |
| Yes | Ref |  |  | Ref |  |
| No | 7.350 (1.440-37.520) | 0.016 |  | 14.933 (1.442-154.649) | 0.023 |

| **Supplementary Table S5: Type A multivariate Cox regression models investigating the factors associated with OS and DSS for patients treated without RAI therapy (N=42)** | | | | | |
| --- | --- | --- | --- | --- | --- |
| **Variables** | **OS** | |  | **DSS** | |
|  | **HR (95%CI)** | ***P*** |  | **HR (95%CI)** | ***P*** |
| **Age at diagnosis** |  |  |  |  |  |
| <55 | Ref |  |  | Ref |  |
| ≥55 | 20.719 (1.245-344.674) | 0.035 |  | 8.145 (1.201-55.219) | 0.032 |
| **Sex** |  |  |  |  |  |
| Female | Ref |  |  | Ref |  |
| Male | 5.833 (1.198-28.397) | 0.029 |  | 2.284 (0.616-8.475) | 0.217 |
| **Race** |  |  |  |  |  |
| White | Ref |  |  | Ref |  |
| Black | 0.328 (0.009-12.069) | 0.545 |  | 0.877 (0.055-13.943) | 0.926 |
| Other | 1.065 (0.071-16.002) | 0.964 |  | 0.573 (0.049-6.648) | 0.656 |
| **Tumor size** |  |  |  |  |  |
| ≤4 cm | Ref |  |  | Ref |  |
| >4 cm | 2.342 (0.545-2.004) | 0.253 |  | 1.730 (0.403-7.407) | 0.461 |
| **Multifocal** |  |  |  |  |  |
| No | Ref |  |  | Ref |  |
| Yes | 1.323 (0.065-26.980) | 0.856 |  | 3.796 (0.425-33.897) | 0.744 |
| Unknown | 2.769 (0.258-29.689) | 0.400 |  | 1.126 (0.186-6.800) | 0.598 |
| **Extrathyroidal extension** |  |  |  |  |  |
| No | Ref |  |  | Ref |  |
| Yes | 7.910 (1.348-46.428) | 0.022 |  | 4.586 (0.908-23.162) | 0.065 |
| **Surgery of thyroid gland** |  |  |  |  |  |
| Lob | Ref |  |  | Ref |  |
| TT | 0.114 (0.008-1.653) | 0.142 |  | 0.696 (0.081-5.954) | 0.741 |
| **Radiation** |  |  |  |  |  |
| EBRT | Ref |  |  | Ref |  |
| No evidence | 5.086 (0.534-48.416) | 0.157 |  | 1.668 (0.303-9.185) | 0.557 |
| **CCND** |  |  |  |  |  |
| Yes | Ref |  |  | Ref |  |
| No | 3.951 (0.803-19.945) | 0.082 |  | 2.977 (0.786-15.539) | 0.095 |

| **Supplementary Table S6: Type B multivariate Cox regression models investigating the factors associated with OS and DSS for patients treated without RAI therapy (N=42)** | | | | | |
| --- | --- | --- | --- | --- | --- |
| **Variables** | **OS** | |  | **DSS** | |
|  | **HR (95%CI)** | ***P*** |  | **HR (95%CI)** | ***P*** |
| **Age at diagnosis** |  |  |  |  |  |
| <55 | Ref |  |  | Ref |  |
| ≥55 | 3.873 (1.101-13.621) | 0.035 |  | 2.360 (0.787-7.077) | 0.125 |
| **Sex** |  |  |  |  |  |
| Female | Ref |  |  | Not Included | |
| Male | 2.075 (0.741-5.811) | 0.165 |  |  |  |
| **Extrathyroidal extension** |  |  |  |  |  |
| No | Ref |  |  | Ref |  |
| Yes | 3.094 (1.115-8.587) | 0.030 |  | 2.554 (0.965-6.760) | 0.059 |
| **CCND** |  |  |  |  |  |
| Yes | Ref |  |  | Ref |  |
| No | 3.972 (1.103-14.155) | 0.035 |  | 3.981 (0.889-17.835) | 0.071 |

| **Supplementary Table S7: Type A multivariate Cox regression models investigating the factors associated with OS and DSS for patients with T3/T4 stage (N=77)** | | | | | |
| --- | --- | --- | --- | --- | --- |
| **Variables** | **OS** | |  | **DSS** | |
|  | **HR (95%CI)** | ***P*** |  | **HR (95%CI)** | ***P*** |
| **Age at diagnosis** |  |  |  |  |  |
| <55 | Ref |  |  | Ref |  |
| ≥55 | 6.571 (1.591-27.131) | 0.009 |  | 6.145 (0.895-42.198) | 0.065 |
| **Sex** |  |  |  |  |  |
| Female | Ref |  |  | Ref |  |
| Male | 2.519 (0.825-7.696) | 0.105 |  | 2.062 (0.471-9.032) | 0.337 |
| **Race** |  |  |  |  |  |
| White | Ref |  |  | Ref |  |
| Black | 0.597 (0.113-3.155) | 0.544 |  | 1.594 (0.279-9.098) | 0.600 |
| Other | 0.486 (0.046-5.164) | 0.549 |  | 0.398 (0.031-5.119) | 0.480 |
| **Tumor size** |  |  |  |  |  |
| ≤4 cm | Ref |  |  | Ref |  |
| >4 cm | 2.268 (0.391-13.158) | 0.362 |  | 0.835 (0.113-6.197) | 0.860 |
| **Multifocal** |  |  |  |  |  |
| No | Ref |  |  | Ref |  |
| Yes | 4.264 (1.088-16.715) | 0.037 |  | 7.431 (1.246-44.327) | 0.028 |
| Unknown | 2.861 (0.683-11.982) | 0.150 |  | 1.443 (0.241-8.641) | 0.688 |
| **Extrathyroidal extension** |  |  |  |  |  |
| No | Ref |  |  | Ref |  |
| Yes | 2.948 (1.039-8.368) | 0.042 |  | 7.382 (1.546-35.252) | 0.012 |
| **Surgery of thyroid gland** |  |  |  |  |  |
| Lob | Ref |  |  | Ref |  |
| TT | 0.441 (0.076-2.559) | 0.362 |  | 0.313 (0.029-3.435) | 0.342 |
| **Radiation** |  |  |  |  |  |
| RAI | Ref |  |  | Ref |  |
| EBRT | 0.654 (0.134-3.206) | 0.601 |  | 1.514 (0.204-11.217) | 0.685 |
| No evidence | 1.310 (0.371-4.620) | 0.675 |  | 6.047 (0.976-37.453) | 0.053 |
| **CCND** |  |  |  |  |  |
| Yes | Ref |  |  | Ref |  |
| No | 3.659 (1.196-11.196) | 0.023 |  | 4.414 (1.084-17.967) | 0.038 |

| **Supplementary Table S8: Type B multivariate Cox regression models investigating the factors associated with OS and DSS for patients with T3/T4 stage (N=77)** | | | | | |
| --- | --- | --- | --- | --- | --- |
| **Variables** | **OS** | |  | **DSS** | |
|  | **HR (95%CI)** | ***P*** |  | **HR (95%CI)** | ***P*** |
| **Age at diagnosis** |  |  |  |  |  |
| <55 | Ref |  |  | Ref |  |
| ≥55 | 4.049 (1.278-12.831) | 0.017 |  | 3.075 (0.704-13.429) | 0.135 |
| **Sex** |  |  |  |  |  |
| Female | Ref |  |  | Not Included | |
| Male | 1.903 (0.739-4.896) | 0.182 |  |  |  |
| **Multifocal** |  |  |  |  |  |
| No | Ref |  |  | Ref |  |
| Yes | 3.504 (1.027-11.957) | 0.045 |  | 4.045 (0.940-17.410) | 0.061 |
| Unknown | 2.715 (0.789-9.341) | 0.113 |  | 2.285 (0.481-10.861) | 0.299 |
| **Extrathyroidal extension** |  |  |  |  |  |
| No | Ref |  |  | Ref |  |
| Yes | 3.168 (1.270-7.901) | 0.013 |  | 4.706 (1.351-16.395) | 0.015 |
| **CCND** |  |  |  |  |  |
| Yes | Ref |  |  | Ref |  |
| No | 3.318 (1.107-9.946) | 0.032 |  | 4.178 (1.040-16.780) | 0.044 |

| **Supplementary Table S9: Baseline characteristics of patients with T1/T2 tumors (N=35)** | | | | | |
| --- | --- | --- | --- | --- | --- |
| **Characteristics** | **Lob/TT+CCND group N=12** |  | **Lob/TT group N=23** |  | ***P*** |
| **Age at diagnosis** |  |  |  |  | 0.713 |
| Median (Range) | 54 (27-82) |  | 59 (21-86) |  |  |
| <55 | 6 (50.0%) |  | 10 (43.5%) |  |  |
| ≥55 | 6 (50.0%) |  | 13 (56.5%) |  |  |
| **Sex** |  |  |  |  | 0.089 |
| Female | 11 (91.7%） |  | 15 (65.2%) |  |  |
| Male | 1 (8.3%) |  | 8 (34.8%) |  |  |
| **Race** |  |  |  |  | 0.419 |
| White | 11 (91.7%) |  | 18 (78.3%) |  |  |
| Black | 1 (8.3%) |  | 2 (8.7%) |  |  |
| Other | 0 (0.0%) |  | 3 (13.0%) |  |  |
| **Multifocal** |  |  |  |  | 0.573 |
| No | 9 (75.0%) |  | 16 (69.6%) |  |  |
| Yes | 3 (25.0%) |  | 5 (21.7%) |  |  |
| Unknown | 0 (0.0%) |  | 2 (8.7%) |  |  |
| **Surgery of thyroid gland** |  |  |  |  | 0.722 |
| Lob | 2 (16.7%) |  | 5 (21.7%) |  |  |
| TT | 10 (83.3%) |  | 18 (78.3%) |  |  |
| **Radiation** |  |  |  |  | 0.756 |
| RAI | 9 (75.0%) |  | 17 (73.9%) |  |  |
| EBRT | 0 (0.0%) |  | 1 (4.3%) |  |  |
| No evidence | 3 (25.0%) |  | 5 (21.7%) |  |  |
